# Genome Comparison of an Ancestral Isolate and a Modern Isolate of *Mycobacterium tuberculosis* of the Beijing Lineage from São Paulo, Brazil

**DOI:** 10.1128/genomeA.01129-15

**Published:** 2015-10-01

**Authors:** Lia Lima Gomes, Michel Abanto Marin, Elena Lasunskaia, Sidra Ezidio Gonçalves Vasconcellos, Marcelo Emanuel Ivens de Araujo, Antonio Basílio de Miranda, Philip Noel Suffys

**Affiliations:** a Laboratory of Molecular Biology Applied to Mycobacteria, Oswaldo Cruz Institute, Oswaldo Cruz Foundation, Rio de Janeiro, Brazil; b Laboratory of Molecular Genetics of Microorganisms, Oswaldo Cruz Institute, Oswaldo Cruz Foundation, Rio de Janeiro, Brazil; c Laboratory of Biology of Recognition, Universidade Estadual do Norte Fluminense, Campos, Rio de Janeiro, Brazil; d Pole Computational Biology and Systems, Oswaldo Cruz Institute, Oswaldo Cruz Foundation, Rio de Janeiro, Brazil

## Abstract

*Mycobacterium tuberculosis* of the Bejing subtype (*Mtb*B) is transmitted efficiently in high burden countries for this genotype. A higher virulence was associated with isolates of the “modern” Beijing genotype sub-lineages when compared to “ancient” ones. Here, we report the full genomes of the strain representing these two genotypes from Brazil, a country with a low incidence of *Mtb*B.

## GENOME ANNOUNCEMENT

Tuberculosis (TB), mainly caused by *Mycobacterium tuberculosis* (*Mtb),* is a serious public health problem in Brazil*.* One particular set of *Mtb* strains, the strains of Beijing family belonging to lineage 2 (East Asian), is very abundant in some countries and has been responsible for outbreaks in different geographical settings, attributed to high transmission capacity, better adaptation to the host environment, and higher virulence and drug resistance levels. This is not the case in Brazil, where the first isolates of *Mtb* Beijing were detected in 2002 and continue to cause only 0.8% of TB cases (1). Because so-called “modern Beijing strains” were suggested to be more virulent than the ancient ones (2), we evaluated some *Mtb*B strains from Brazil of either genotype in virulence models and observed higher virulence in the modern strains (3).

Here, we report the whole-genome sequences of two *Mtb* Beijing isolates obtained from patients in São Paulo, one with the ancient genotype (ZT272) and the other with the modern genotype (5351), the latter presenting a more virulent phenotype (4). The DNA was extracted using a Charge Switch gDNA bacteria DNA mini kit (Invitrogen) and a paired-end library was prepared using the Nextera XT DNA library kit, followed by 2 × 100-bp sequencing on an Illumina HiSeq2500 sequencer at the High Throughput Sequencing Platform of the Oswaldo Cruz Institute (Fiocruz, Rio de Janeiro, Brazil). Initial quality control of raw reads was performed using fastaQC. Quality trimming, adaptor removal and assembly of high-quality reads (between 36 and 83 reads) was realized by the software A5-miseq (http://sourceforge.net/projects/ngopt/). *De novo* assembly of the reads from ZT272 and 5351 strains yielded, respectively, 255 and 163 contigs, a genome size of 4,347,734 and 4,355,078 bp and an average coverage of 207 and 112×. Respectively, 4,258 and 4,408 open reading frames were detected using RAST server. Alignment*,* annotation, and single nucleotide polymorphism (SNP) and indel calling was performed using Bionumerics software (version 7.5; Applied Maths) and using the genome sequence of *Mtb* H37rv (GenBank accession no. NC_000962.3) as a reference. The SNPs found were grouped by functional category according to the database Tuberculist (http://tuberculist.epfl.ch/). When compared to *Mtb* H37Rv, ancient sp. ZT272 and modern sp. 5351 presented 7,332 and 4,398 SNPs and 922 and 675 indels, respectively. Among the 88 virulence associated genes defined in *Mtb* H37Rv (5), three (*plc*D-*plc*D, *esx*M, and *esx*N) were absent in both strains while in addition, the ancient strain had an also deleted *esp*B and *Rv*3879. When comparing the number of SNP specifically in the virulence genes only, comparing to *Mtb* H37Rv, a larger number of SNPs was observed in the modern strain (41) than in the ancient one (32), with 22 SNPs in common and an additional 19 and 10 unique SNPs, respectively.

Although the overall genome of the ancestral strain seems more distant from the H37Rv strain than the modern one, the contrary is observed in the virulence associated genes and further investigation as to whether this is related with higher virulence is needed.

### Nucleotide sequence accession numbers.

These whole-genome shotgun projects have been deposited at DDBJ/EMBL/GenBank under the accession no. LGTJ00000000 and JXXH00000000. The versions described in this paper are versions LGTJ01000000 and JXXH01000000.
